# Syk Tyrosine Kinase Is Critical for B Cell Antibody Responses and Memory B Cell Survival

**DOI:** 10.4049/jimmunol.1500461

**Published:** 2015-04-10

**Authors:** Jochen A. Ackermann, Josquin Nys, Edina Schweighoffer, Scott McCleary, Nicholas Smithers, Victor L. J. Tybulewicz

**Affiliations:** * Division of Immune Cell Biology, MRC National Institute for Medical Research, London NW7 1AA, United Kingdom; and; † Immuno-Inflammation Therapy Area Unit, GlaxoSmithKline, Stevenage SG1 2NY, United Kingdom

## Abstract

Signals from the BCR are required for Ag-specific B cell recruitment into the immune response. Binding of Ag to the BCR induces phosphorylation of immune receptor tyrosine-based activation motifs in the cytoplasmic domains of the CD79a and CD79b signaling subunits, which subsequently bind and activate the Syk protein tyrosine kinase. Earlier work with the DT40 chicken B cell leukemia cell line showed that Syk was required to transduce BCR signals to proximal activation events, suggesting that Syk also plays an important role in the activation and differentiation of primary B cells during an immune response. In this study, we show that Syk-deficient primary mouse B cells have a severe defect in BCR-induced activation, proliferation, and survival. Furthermore, we demonstrate that Syk is required for both T-dependent and T-independent Ab responses, and that this requirement is B cell intrinsic. In the absence of Syk, Ag fails to induce differentiation of naive B cells into germinal center B cells and plasma cells. Finally, we show that the survival of existing memory B cells is dependent on Syk. These experiments demonstrate that Syk plays a critical role in multiple aspects of B cell Ab responses.

## Introduction

The clonal selection hypothesis proposes that the specificity of the BCR is the critical determinant of whether any given B lymphocyte is recruited into the immune response (1, 2). Ag-induced activation of B cells results in their differentiation into Ab-secreting cells and, for T-dependent responses, into germinal center and memory B cells. During the germinal center reaction, B cells undergo somatic hypermutation resulting in mutation of the BCR, with subsequent selective survival and expansion of B cells whose BCR has a higher affinity for Ag. The selective activation of B cells with Ag-specific BCRs and subsequent selection of cells with BCRs of increased affinity implies that signaling from the BCR plays a crucial role during the Ab response.

The BCR is composed of surface-bound Ig noncovalently associated with nonpolymorphic transmembrane signaling proteins CD79a and CD79b (Ig-α and Ig-β) that contain ITAMs in their cytoplasmic domains (3, 4). Binding of Ag to the BCR results in phosphorylation of two tyrosine residues in the ITAMs of CD79a and CD79b, which then recruit Syk tyrosine kinase via its two SH2 domains, thereby activating it (5). The phosphorylation of the ITAMs is mediated by Src-family kinases, such as Lyn, as well as by Syk itself (6, 7).

The direct binding of Syk to the BCR and its subsequent activation has suggested that it plays an important role in downstream signaling. This was first demonstrated directly in DT40 cells, a chicken B cell leukemia, in which genetic deletion of *Syk* resulted in a complete block in BCR-induced early signaling events such as intracellular Ca^2+^ flux and phosphorylation of phospholipase-Cγ2 (8). Subsequently, analysis of Syk-deficient mice showed that loss of the kinase resulted in a complete block in B cell development, with a partial block at the pro-B to pre-B cell transition and a complete block at the immature to mature B cell transition (9–11). These transitions correspond to points where signals from the pre-BCR or the BCR are required for cells to progress in development, and suggest that the blocks occur because these receptors are unable to signal correctly in the absence of Syk. In support of this suggestion, B cell development is completely arrested at the pre-BCR checkpoint in compound mutant mice lacking both Syk and the related ZAP70 kinase, and when pro-B cells missing these kinases are stimulated with an anti-CD79b Ab, the cells fail to develop into pre-B cells, in contrast to wild type cells (12).

Despite the clear importance of Syk in B cell development, its role in the activation of mature primary B cells during immune responses remains unknown. The lack of B cells in Syk-deficient mice means that it is not possible to use these to study the role of Syk in mature B cells. However, we have recently established a mouse strain with a conditional allele of *Syk*, in which the gene can be inactivated in response to tamoxifen, thereby generating Syk-deficient mature B cells (13). We used this strain to show that Syk is required for B cell survival. Loss of Syk causes many mature B cells to die. However, crucially, ∼20% of the follicular B cells remain and can be used for the study of Syk function in primary B cells.

We show here that Syk is required for in vitro BCR-induced activation of B cells and in vivo for both T-dependent and T-independent Ab responses. We demonstrate that Syk is absolutely required for the differentiation of follicular B cells into germinal center B cells and Ab-secreting plasma cells. Furthermore, by deleting Syk after a primary immunization, we show that the kinase is also required for the secondary memory Ab response. Finally, we show that Syk is required for the survival of memory B cells.

## Materials and Methods

### Mice

Mice carrying a conditional allele of *Syk* (*Syk*^tm1.1Nns^, *Syk*^fl^), a constitutive disruption of *Syk* (*Syk*^tm1Tyb^, *Syk^−^*), an allele of ROSA26 expressing the MerCreMer fusion protein (Gt(ROSA)26Sor^tm1(cre/Esr1*)Nns^, *Rosa26*^MerCreMer^, RMCM), disruption of the IgM gene (*Ighm*^tm1Cgn^, μMT), and a disruption of *Rag1* (*Rag1*^tm1Mom^) have been described previously (10, 13, 14). SWHEL mice expressing an anti–hen egg lysozyme (HEL) BCR were a gift from R. Brink and consisted of a knock-in mutation in the *IgH* locus (*Igh*^tm1Rbr^) and an Ig L chain transgene (Tg(IgkHyHEL10)1Rbr) bred together to generate the SWHEL strain (*Igh*^tm1Rbr/tm1Rbr^Tg(IgkHyHEL10)1Rbr) (15). All strains were bred on a C57BL/6JNimr background. B6.SJL and (B6 x B6.SJL)F1 mice were obtained from the breeding facility at the National Institute for Medical Research. To induce Cre activity, mice were injected i.p. for 5 d with 2 mg/day of tamoxifen (Sigma).

To generate mixed radiation chimeras, bone marrow was harvested from *Syk*^fl/+^RMCM or *Syk*^fl/−^RMCM mice, treated with ACK lysis buffer and mixed in a 1:2 ratio with bone marrow cells from μMT (*Ighm*^tm1Cgn/tm1Cgn^) mice, and injected intravenously at 1 × 10^6^ cells per recipient into Rag1-deficient animals that were irradiated with 5 Gy using a ^137^Cs source. Mice received Baytril in their drinking water (0.02%, Bayer Healthcare) for at least 4 wk after transplantation, and were used for further studies no less than 6 wk after reconstitution.

### Cell culture

Splenic B cells were purified from mice treated 21 d earlier with tamoxifen, by magnetic negative depletion using biotinylated Abs against CD43 (eBioR2/60), CD11c (N418), CD11b (M1/70), and CD3ε (145-2C11; eBioscience) and streptavidin Dynabeads (Life Technologies). B cells (10^7^ cells/ml) were incubated in PBS containing 0.5 μM CFSE (Molecular Probes) at 37°C for 10 min. Cells were washed twice in DMEM-plus medium (DMEM with 10% FCS, 100 U/ml penicillin, 100 μg/ml streptomycin, 100 μM nonessential amino acids, 20 mM HEPES buffer, 2 mM l-glutamine, and 50μM 2-ME) and then cultured at 1–1.5 × 10^6^ cells/ml in DMEM-plus alone or supplemented with 10 μg/ml anti-IgM F(ab)_2_ (Jackson Immunoresearch) or 100 ng/ml CD40L (R&D Systems) and 20–100 ng/ml IL-4 (PeproTech). Flow cytometry was used to assess surface expression of proteins, proliferation by CFSE dilution, and number of live cells by a LIVE/DEAD fixable near-infrared dead cell stain.

### Immunizations

For T-independent responses, mice were immunized i.p. with 10 μg TNP-Ficoll. For T-dependent immune responses, mice were immunized i.p. with 50–100 μg Alum-precipitated (Thermo Scientific) 4-hydroxy-3-nitrophenylacetyl conjugated to chicken-γ-globulin (NP-CGG), with a ratio of NP to CGG ranging from 21 to 27 (BioSearch Technologies). For recall, responses mice were injected i.p. with 50 μg NP-CGG in PBS. TNP or NP-specific Abs in the serum were measured by ELISA using Maxisorp plates (Nunc) coated with NP_18_-BSA as previously described (16).

### Flow cytometry

Single-cell suspensions of splenocytes were treated with ACK lysis buffer to remove RBCs before staining in PBS, containing LIVE/DEAD fixable near-IR dead cell stain (Life Technologies) and appropriate, pretitered Abs. Abs used, indicating Ag and fluorophore (and clone): CD69-FITC (H1.2F3), CD86-PE (GL-1), streptavidin-PerCP (Becton Dickinson), MHC class II-bio (I-Ab; M5/114.15.2), IgM-PECy7 (II/41), B220-eFluor450 (RA3-6B2; eBioscience), CD19-FITC, B220-PerC, IgM-PE, IgG1-APC, PNA-FITC, CD38-PE/Cy7, CD45.2-ef450, CD45.1-FITC, CD138-PE, IgD-ef450, CD4-FITC, CD8-FITC, CD45.2-PerCP-cy5.5, CD45.1-FITC, B220-BV605, CD4-BV650, CD8a-BV650, CD38-bio, Streptavidin-PacO, FAS-PE. Anti-Syk Ab (5F5; BioLegend) and NIP-BSA were labeled using LYNX Rapid APC, RPE, and PerCP Ab Conjugation Kits (AbD Serotec). Ab staining of intracellular Syk was performed as described previously (13).

### ELISPOT

Nitrocellulose 96-well filtration plates (Millipore) were coated overnight at 4°C with 50 μg/ml NP_18_-BSA (Biosearch Technologies) and then blocked with DMEM. Splenocytes or bone marrow cells were serially diluted (maximum concentration of 4 × 10^5^ cells/well) on the coated plates and cultivated overnight in DMEM-plus with 5% FCS at 37°C, 5% CO_2_. Plates were washed twice with PBS, 0.05% Tween-20 and twice with PBS, stained with biotinylated anti-IgG1 or anti-IgM Abs for 2 h at room temperature, washed four times with PBS-Tween, and stained with Avidin d-alkaline phosphatase for 1 h at room temperature. Alkaline phosphatase activity was visualized using BCIPT/NPT substrate (BioFX). Spots, each representing a single Ab-secreting cell, were counted using an ImmunoSpot reader.

### Cell transfers and immunization with HEL

To induce primary responses 2.5 × 10^4^ HEL-binding B cells from tamoxifen-treated SWHEL/*Syk*^fl/fl^ or SWHEL/*Syk*^fl/fl^RMCM mice were transferred into B6.SJL recipient mice together with 5 × 10^8^ HEL-conjugated sheep RBCs (SRBC). To generate a memory pool, splenocytes containing an equivalent of 5 × 10^5^ HEL-binding B cells from SWHEL/*Syk*^fl/+^RMCM or SWHEL/*Syk*^fl/fl^RMCM mice were transferred into (B6 x B6.SJL)F1 recipient mice together with 1 × 10^9^ HEL^3×^-conjugated SRBC. HEL (Sigma-Aldrich) or HEL^3×^ (a gift from R. Brink) (17) was conjugated to SRBC (Patricell) as described previously (18). The germinal center reaction was blocked by three injections of 300 μg anti-CD40L (MR1) or control Hamster IgG (BioXcell) every second day.

### Statistical analysis

All statistical comparisons used the nonparametric two-tailed Mann–Whitney *U* test or Student *t* test. Statistically significant differences are indicated in the figures.

## Results

### Syk is required for in vitro BCR-induced activation

**FIGURE 1.**
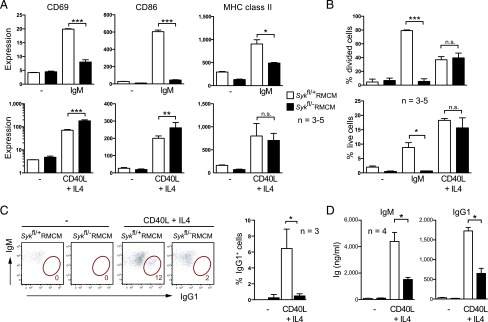
Syk is required for activation of B cells in response to anti-IgM and class switching in response to CD40L + IL-4. (**A**) Mean (±SEM) expression of CD69, CD86, and MHC class II on B cells of the indicated genotypes stimulated with anti-IgM or CD40L plus 100ng/ml IL-4 or left unstimulated (−) for 36 h. (**B**) Mean (±SEM) percentage of divided cells and percentage of live cells in cultures of B cells stimulated as in (A) for 60 h. (**C**) Dot plots of surface staining of IgM and IgG1 on B cells of the indicated genotypes stimulated for 60 h with CD40L plus 20 ng/ml IL-4 or left unstimulated (−). Numbers indicate the percentage of cells falling into the gate. Graph of mean (±SEM) percentage of IgG1^+^ B cells in cultures, determined as in dot plots. (**D**) Mean (±SEM) levels of IgM and IgG1 secreted into the medium of B cells cultured as in (C) for 6 d. *p < 0.05, **p < 0.01, ***p < 0.001. n.s., not significant.

Initially, we investigated whether Syk was required for Ag receptor-induced activation of B cells in vitro, using mice containing a conditional allele of *Syk* in which exon 11 is flanked by loxP sites (*Syk*^fl^) and a tamoxifen-inducible Cre recombinase expressed from the ROSA26 locus (*Rosa26*^MerCreMer^, RMCM) (13). These strains and mice bearing a deleted allele of Syk (*Syk^−^*) (10) were intercrossed to generate control (*Syk*^fl/+^RMCM) and conditional mutant (*Syk*^fl/^*^−^*RMCM) mice. Treatment with tamoxifen resulted in deletion of the *Syk*^fl^ allele in both strains, leaving control and mutant mice with either one or no functional alleles of *Syk*. As established previously, by 10 d after tamoxifen treatment, Syk protein is no longer detectable in splenic B cells from mutant mice, and by 21 d ∼80% of the B cells are lost because of impaired survival (13). Despite this loss of B cells, ∼20% of follicular B cells persist in the mutant but have no detectable Syk. Thus, we were able to use these Syk-deficient B cells to investigate whether Syk was required for BCR-induced activation in vitro. We found that Syk was required for BCR-induced increase in cell surface expression of CD69, CD86, and MHC class II proliferation and survival (Fig. 1A, 1B). In contrast, Syk-deficient B cells were able to respond at least as well as control cells to stimulation with CD40L plus IL-4, as measured by the same parameters, demonstrating that Syk-deficient cells retained the capacity to become activated and to proliferate. However, Ig isotype class switching to IgG1 and secretion of IgM and IgG1 in response to CD40L plus IL4 were greatly reduced in the absence of Syk (Fig. 1C, 1D). Thus, Syk was required in all BCR-induced in vitro activation responses we measured, but only in a select subset of responses induced by CD40L plus IL-4.

### Defective T-independent responses in the absence of Syk

**FIGURE 2.**
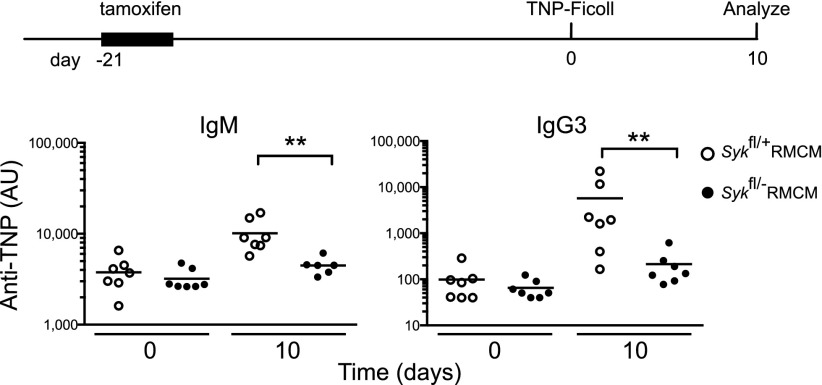
Defective T-independent Ab response in the absence of Syk in B cells. Irradiated Rag1-deficient mice reconstituted with a mixture of μMT (B cell–deficient) bone marrow and Syk-expressing (*Syk*^fl/+^RMCM) or Syk-deficient (*Syk*^fl/−^RMCM) bone marrow were treated with tamoxifen, immunized 21 d later with TNP-Ficoll, and analyzed 10 d later. Graphs show levels of anti-TNP IgM or IgG3 in the serum of the mice (dots, individual mice; line, mean). **p < 0.01. AU, arbitrary units.

Next, we investigated the requirement for Syk during in vivo B cell responses to Ag. The *Rosa26*^MerCreMer^ allele is expressed ubiquitously; therefore, to rule out secondary effects on Ab responses because of deletion of Syk in non–B cells, we limited the loss of Syk to B cells by generating mixed chimeras in which the hematopoietic system of irradiated Rag1-deficient mice was reconstituted with a mixture of bone marrow from μMT (B cell–deficient) mice and either Syk-expressing (*Syk*^fl/+^RMCM) or Syk-deficient (*Syk*^fl/−^RMCM) mice. In the resulting mutant mice, all B cells lost Syk expression after tamoxifen treatment, whereas Syk was maintained in other cell types. Initially, we determined whether Syk was required for T-independent type-II (TI-II) responses to TNP-Ficoll, a hapten coupled to highly repetitive polysaccharide, by immunizing chimeric mice 21 d after treatment with tamoxifen. We found that in the absence of Syk, there was a significant reduction in Ag-specific IgM and IgG3 (Fig. 2), demonstrating a B cell-intrinsic requirement for Syk in TI-II Ab responses.

### Defective T-dependent Ab response in the absence of Syk in B cells

**FIGURE 3.**
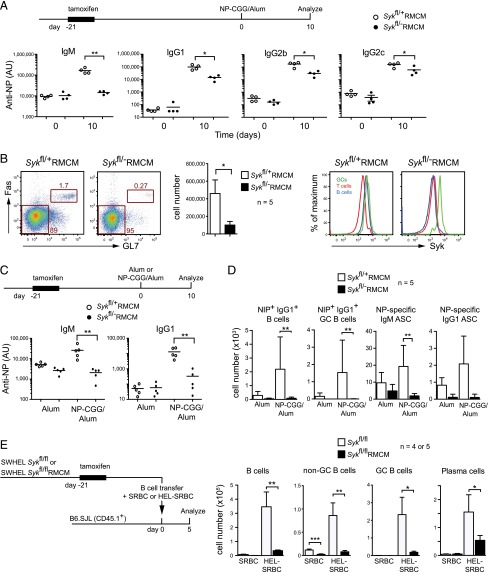
Defective T-dependent Ab response in the absence of Syk in B cells. (**A** and **B**) Syk-expressing (*Syk*^fl/+^RMCM) or Syk-deficient (*Syk*^fl/−^RMCM) mice were treated with tamoxifen, immunized 21 d later with NP-CGG in Alum, and analyzed 10 d later. (A) Graph shows anti-NP Ig of the indicated isotype in serum of mice of the indicated genotypes either just before immunization (day 0) or 10 d later (dots, individual mice; line, mean). (B) Dot plots of splenocytes from mice 10 d after immunization gated on B220^+^ cells showing staining with Abs to Fas and GL7. Numbers indicate the percentage of cells falling into each gate. Column graph shows mean (±SEM) number of germinal center B cells (Fas^+^GL7^+^) determined as in dot plots. Histograms show levels of Syk in T cells (B220^−^), B cells (B220^+^) and germinal center B cells (GCs). T cells show no detectable Syk by this assay. (**C** and **D**) Irradiated Rag1-deficient mice reconstituted with a mixture of μMT bone marrow and Syk-expressing (*Syk*^fl/+^RMCM) or Syk-deficient (*Syk*^fl/−^RMCM) bone marrow were treated with tamoxifen, immunized 21 d later with NP-CGG in alum or alum alone, and analyzed 10 d after that. (C) Graph of anti-NP IgM or IgG1 in mice of the indicated genotypes (dots, individual mice; line, mean). (D) Mean (±SEM) number of Ag-specific switched B cells (B220^+^NIP^+^IgG1^+^) or germinal center B cells (B220^+^Fas^+^GL7^+^NIP^+^IgG1^+^; Supplemental Fig. 1A) or Ab-secreting cells (ASCs) making NP-specific IgM or IgG1. (**E**) SWHEL *Syk*^fl/fl^ or *Syk*^fl/fl^RMCM mice were treated with tamoxifen and 21 d later B cells were transferred into B6.SJL mice. Recipient mice were immunized with SRBC or HEL-SRBC and analyzed 5 d later. Graphs show mean (±SEM) number of total splenic donor-derived B cells, non-GC B cells, GC B cells, and plasma cells (Supplemental Fig. 1B). *p < 0.05, **p < 0.01, ***p < 0.001.

To determine whether Syk was also required for T-dependent B cell responses, *Syk*^fl/+^RMCM and *Syk*^fl/−^RMCM mice were treated with tamoxifen, immunized 21 d later with NP-CGG precipitated in alum, and then analyzed after an additional 10 d (Fig. 3A). We found that loss of Syk resulted in a significant decrease in Ag-specific IgM, IgG1, IgG2b, and IgG2c in the serum, and in the induction of far fewer germinal center B cells (Fig. 3B). Notably, although most B cells in the mutant mice had no detectable Syk expression, the few remaining germinal center B cells in the same mice still expressed Syk (Fig. 3B). This strong selection against cells that have deleted Syk indicates that B cells require Syk to differentiate into germinal center cells.

To evaluate whether the requirement for Syk in T-dependent B cell responses was B cell-intrinsic, we immunized chimeric mice with NP-CGG. We found that restricted loss of Syk in B cells resulted in a large decrease in Ag-specific IgM and IgG1 in the serum (Fig. 3C), and a large reduction in the numbers of Ag-specific IgG1-expressing B cells and germinal center B cells, as well as a reduction in Ab-secreting cells producing Ag-specific IgM or IgG1 (Fig. 3D).

The reduced T-dependent B cell response could have been caused by a requirement for Syk in B cells to differentiate into germinal center and plasma cells. Alternatively, it could have resulted from Syk deficiency reducing the number of naive B cells, leading to a lower number of precursor cells able to respond to the immunizing Ag. To address this issue, we bred the conditional Syk mutation to SWHEL mice expressing a BCR-specific for HEL (15). In this strain, a recombined VDJ region from a HEL-specific Ab has been inserted into the IgH locus, and the mice also contain a transgene expressing an Igκ L chain from the same Ab. We transferred equal numbers of control and Syk-deficient SWHEL B cells into wild-type recipients, and immunized them with HEL-coupled SRBC or SRBC alone (Fig. 3E). Analysis 5 d later showed that control B cells expanded strongly in mice that were immunized with HEL-SRBC compared with SRBC alone, but that this did not occur in the absence of Syk (Fig. 3E). Subdividing these B cells into nongerminal center and germinal center B cells showed the same result—a large expansion of control Syk-expressing B cells, but not of mutant Syk-deficient cells. Ag-induced expansion of plasma cells was also strictly Syk-dependent. Therefore, B cell–expressed Syk is required for the Ag-induced expansion of B cells and their differentiation into germinal center and plasma cells in response to a T-dependent Ag.

### Defective Ab recall response in the absence of Syk in B cells

**FIGURE 4.**
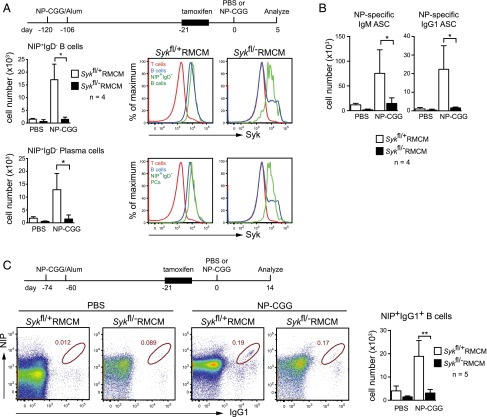
Defective Ab recall response in the absence of Syk in B cells. (**A** and **B**) Irradiated Rag1-deficient mice reconstituted with a mixture of μMT bone marrow and Syk-expressing (*Syk*^fl/+^RMCM) or Syk-deficient (*Syk*^fl/−^RMCM) bone marrow were immunized twice with NP-CGG in alum. Eighty-five days after the second immunization, the mice were treated with tamoxifen; 21 d later they were immunized with NP-CGG in PBS or PBS alone and analyzed 5 d after that. Graphs show mean (±SEM) numbers of Ag-specific switched B cells (NIP^+^IgD^−^; a mix of memory, germinal center, and plasma cells) and plasma cells in the spleen (Supplemental Fig. 2). Histograms show levels of Syk in T cells, all B cells, and Ag-specific switched B cells or plasma cells (PCs). (B) Mean (±SEM) numbers of splenic Ab-secreting cells (ASCs) making NP-specific IgM or IgG1. (**C**) Mice were immunized twice with NP-CGG in alum as in (A); 39 d later they were treated with tamoxifen and 21 d later were challenged with NP-CGG in PBS or PBS alone. Dot plots show binding of Ag (NIP) and expression of surface IgG1 on splenic B cells (B220^+^). Numbers indicate the percentage of cells falling into gate. The graph shows mean (±SEM) numbers of Ag-specific IgG1-expressing B cells (B220^+^NIP^+^IgG1^+^). *p < 0.05, **p < 0.01.

A characteristic feature of the adaptive immune response to T-dependent Ags is a secondary, or recall, response that is typically faster and stronger than the primary response. This is due to the formation of memory B and T cells during the primary response, which respond more rapidly and more strongly to Ag than naive lymphocytes (19). To evaluate whether Syk is also required by memory B cells for their response to Ag, we immunized chimeric mice in which deletion of Syk could be limited to the B cell lineage, but that had not yet been treated with tamoxifen. The mice were immunized twice with NP-CGG precipitated in Alum, treated with tamoxifen 53 or 99 d later to delete Syk in B cells, and given a secondary challenge of NP-CGG in PBS or just PBS alone a further 21d later (Fig. 4A). Analysis of splenocytes 5 d after this secondary boost showed that although the number of control Ag-specific switched B cells or plasma cells increased greatly in response to the secondary challenge with NP-CGG, in the absence of Syk this expansion was largely absent (Fig. 4A). Notably, the few switched B cells or plasma cells seen in these mice still expressed Syk, showing again a strong selection against the loss of Syk in responding B cells. Further analysis showed similar large reductions in Ab-secreting cells making NP-specific IgM and IgG1 and in Ag-specific switched IgG1^+^ B cells (Fig. 4B, 4C). These results indicate that the absence of Syk in B cells greatly diminishes the secondary recall response.

### Syk is required for the survival of memory B cells

**FIGURE 5.**
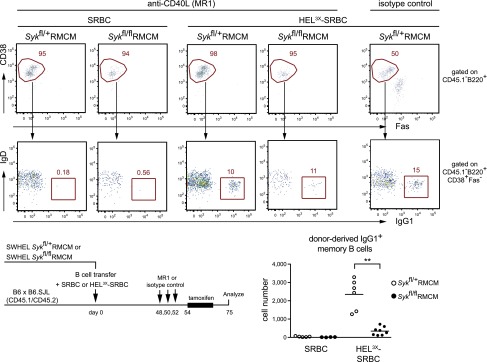
Syk is required for the survival of memory B cells. B cells from SWHEL *Syk*^fl/+^RMCM or *Syk*^fl/fl^RMCM mice were transferred into (B6 x B6.SJL) F1 mice, and recipient mice were immunized with SRBC or HEL^3×^-SRBC. After 48 d, mice were given three injections of anti-CD40L (MR1) or an isotype control Ab and then tamoxifen 54 d after immunization, and they were analyzed 21 d later. Dot plots in the *top row* show expression of CD38 and Fas on donor-derived splenic B cells (CD45.1−B220^+^), and the gate indicates naive and memory B cells (CD38^+^Fas^−^); these in turn were analyzed for expression of IgD and IgG1 in the *lower dot plots*, where the gate shows donor-derived IgG1^+^ memory B cells. Numbers indicate the percentage of cells falling into the gate. The graph shows the number of splenic donor-derived IgG1^+^ memory B cells, with each dot representing a single mouse and the line showing the mean. **p < 0.01.

This requirement for Syk in the secondary T-dependent response could have been due to a role for Syk within memory B cells for their proliferation and differentiation into plasma cells. Alternatively, Syk could have been required for the survival of memory B cells. We have previously shown that the survival of naive B cells is dependent on Syk, in part because it transduces survival signals from BAFFR and the BCR (13). However the signals controlling the survival of memory B cells are largely unknown. It has been established that BAFF is not required for their survival (20), but it is not known whether the BCR is required. Thus, we next investigated the potential role for Syk in memory B cell survival. We again made use of SWHEL mice bred to either control or conditional Syk mutant mice and transferred equal numbers of SWHEL B cells from each strain into wild-type recipients, and immunized with HEL^3×^-SRBC or SRBC alone (Fig. 5). HEL^3×^ is a variant of HEL with a lower affinity for the anti-HEL BCR in SWHEL mice that gives longer-lasting immune responses (17). The donor mice were not treated with tamoxifen; therefore, the SWHEL B cells from both control and conditional mutant mice still expressed Syk and thus could mount a strong immune response, including formation of similar numbers of memory B cells (not shown). Fifty-four days after immunization, the mice were treated with tamoxifen to delete Syk in the conditional mutant memory B cells, and the numbers of these cells was evaluated 21 d later. Importantly, just before treatment with tamoxifen, the mice were given three injections of an anti-CD40L Ab to block the germinal center reaction. Because some germinal center B cells differentiate into memory B cells, and Syk is required for the formation of germinal center B cells, we needed to ensure that any potential drop in memory B cell numbers following deletion of Syk was not due to the loss of germinal center cells. The anti-CD40L treatment effectively eliminated all germinal center cells (compared with treatment with isotype control Ab; Fig. 5). Analysis of memory B cell numbers in mice immunized with HEL^3×^-SRBC showed that deletion of Syk resulted in a large reduction in the number of IgG1^+^ memory B cells (Fig. 5). Thus, Syk is required for the survival of memory B cells.

## Discussion

Our results establish that Syk is a key signal transducer that is required for coupling the BCR to all downstream signaling pathways in mature B cells. All measures of activation including upregulation of cell surface markers, proliferation, and survival were dependent on Syk expression. In contrast, Syk-deficient B cells could be efficiently activated by CD40L plus IL-4, as measured by the same parameters, although class switching was defective, suggesting that Syk must contribute in some way to this process. We have shown previously that survival signals from BAFFR are partially transduced via the BCR to the activation of Syk (13). By analogy, signals from CD40 or IL-4R may be transduced in part via the BCR and Syk. We note that both CD40 and BAFFR are members of the TNFR family and signal by similar mechanisms.

Our work also demonstrates that Syk is absolutely required for both TI-II and T-dependent Ab responses. In particular, Syk-deficient B cells cannot differentiate into germinal center or plasma cells, suggesting that BCR-derived signals transduced via Syk are essential for these differentiation events. A recent study showed that LPS upregulation of the Blimp1 transcription factor in B cells in vitro requires Syk expression (21). Blimp1 is required for full differentiation into plasma cells (22). Our results therefore raise the possibility that the LPS receptor TLR4 also transduces signals via the BCR and Syk to induce Blimp1 transcription.

Finally, we showed that Syk is needed for the survival of memory B cells, a process about which little is known. This requirement for Syk suggests that an ITAM-bearing receptor is also likely to be involved in memory B cell survival, most probably the BCR. However, because Ag is dispensable for memory B cell survival (23), the survival function of Syk may be activated by a BCR that has not been engaged by Ag. We previously showed that Syk is required for the survival of naive follicular B cells, because key survival signals from BAFFR are transduced in part via the BCR to the activation of Syk (13). By analogy, a similar pathway may also operate in memory B cells. However the cytokines BAFF and APRIL are not required for the survival of memory B cells (20), suggesting that if such a BCR/Syk survival pathway operates in memory B cells, it may be downstream of a different cytokine receptor.

## Supplementary Material

Data Supplement
