# Supplementary material for: Syk Tyrosine Kinase Is Critical for B Cell Antibody Responses and Memory B Cell Survival
Source: J Immunol. 2015 Apr 10;194(10):4650–6. doi: 10.4049/jimmunol.1500461 (PMC4416743; doi:10.4049/jimmunol.1500461)
Supplement: Data Supplement [file JI_1500461.zip › JI_1500461_Supplemental_Figures_1.pdf]

Syk tyrosine kinase is critical for B cell antibody responses and memory B cell survival

Jochen A. Ackermann*, Josquin Nys*, Edina Schweighoffer*, Scott McCleary†, Nicholas Smithers†, Victor L. J. Tybulewicz*

*Division of Immune Cell Biology, MRC National Institute for Medical Research, London NW7 1AA, UK; †Immuno-Inflammation Therapy Area Unit, GlaxoSmithKline, Stevenage, UK.

Supplemental Figures

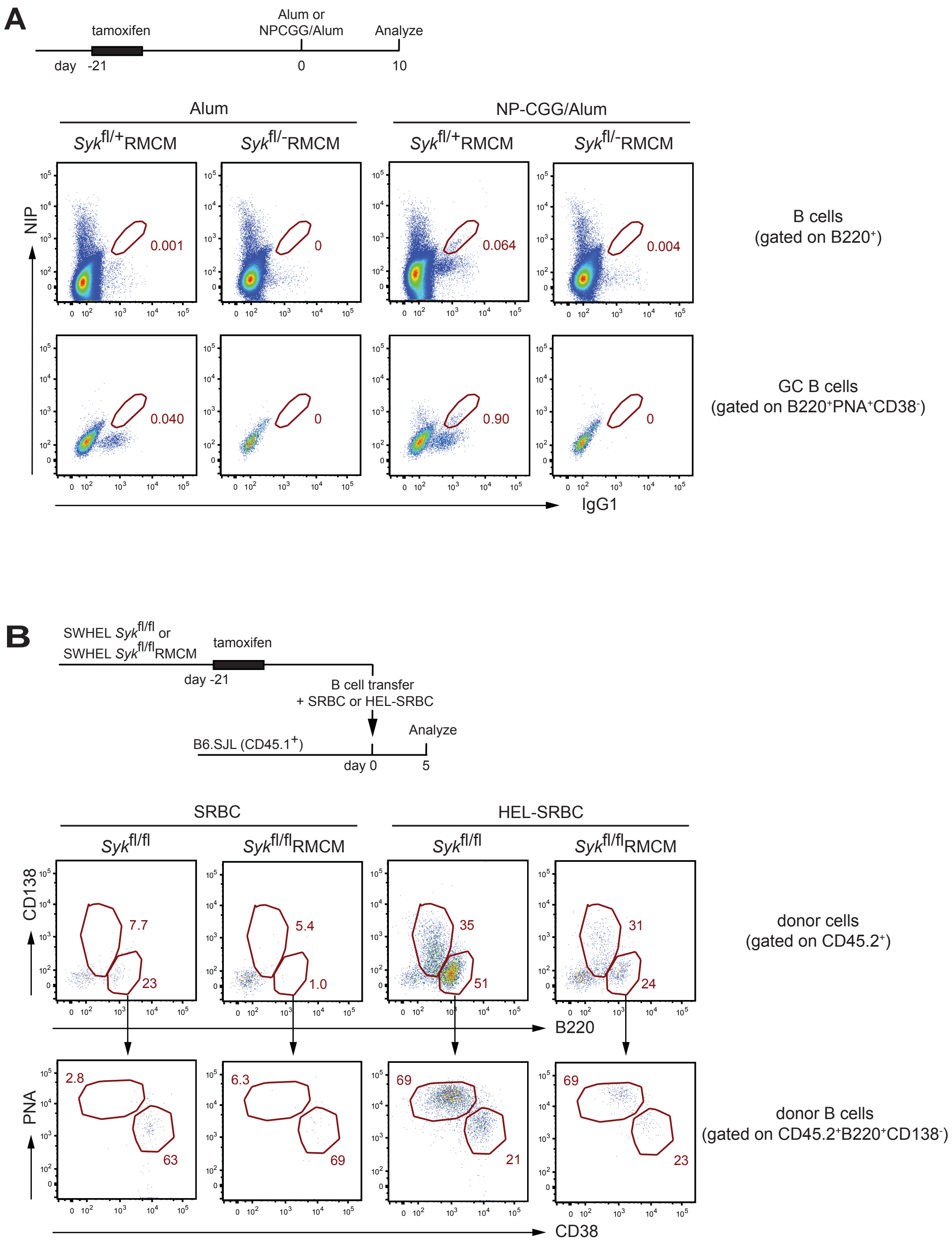

Supplemental Figure 1. Defective T-dependent antibody response in the absence of Syk in B cells. (A) Irradiated Rag1-deficient mice reconstituted with a mixture of μMT bone marrow and Syk-expressing (*Syk*$^{fl/+}$RMCM) or Syk-deficient (*Syk*$^{fl/-}$RMCM) bone

marrow were treated with tamoxifen, immunized 21d later with NP-CGG in Alum or Alum alone and analyzed 10d after that, as shown in the time-line. Dot plots show binding of antigen (NIP) and expression of IgG1 on the surface of splenic B cells or germinal center (GC) B cells. Gates indicate antigen-specific switched cells ($NIP^{+}IgG1^{+}$). Numbers indicate % of cells falling into gate and were used to determine data shown in Fig 3D. (B) SWHEL $Syk^{fl/fl}$ or SWHEL $Syk^{fl/fl}$RMCM mice were treated with tamoxifen and 21d later B cells were transferred into B6.SJL mice, recipient mice were immunized with SRBC or HEL-SRBC and analyzed 5d later, as shown in the time-line. Dot plots in top row show expression of B220 and CD138 on donor ($CD45.2^{+}$) splenocytes and gates indicate B cells ($B220^{+}CD138^{-}$) and plasma cells ($B220^{-}CD138^{+}$). Dot plots in lower row indicate surface binding of PNA and expression of CD38 on donor-derived splenic B cells ($CD45.2^{+}B220^{+}CD138^{-}$). Gates indicate non-germinal center ($PNA^{low}CD38^{+}$) and germinal center ($PNA^{+}CD38^{-}$) cells. Numbers indicate % of cells falling into gate and were used to determine data shown in Fig 3E.

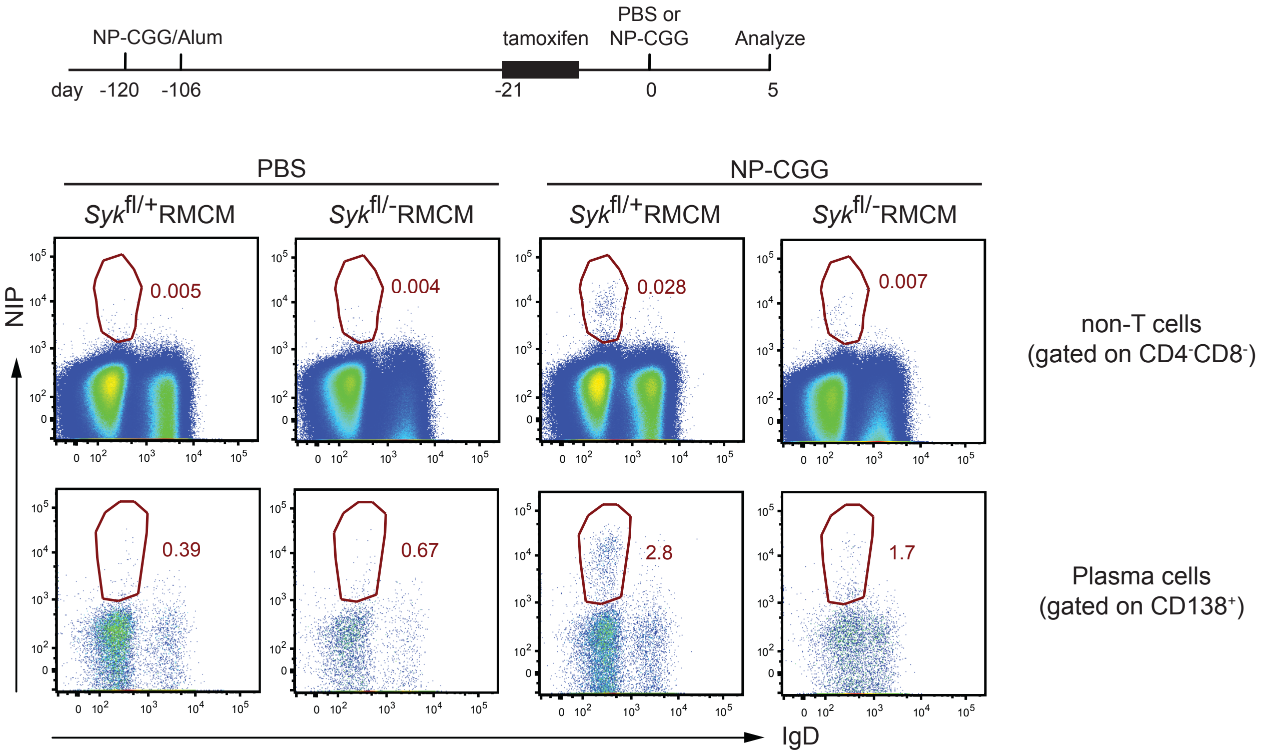

Supplemental Figure 2. Defective antibody recall response in the absence of Syk in B cells. Irradiated Rag1-deficient mice reconstituted with a mixture of µMT bone marrow and Syk-expressing (*Syk*$^{fl/+}$RMCM) or Syk-deficient (*Syk*$^{fl/-}$RMCM) bone marrow were immunized twice with NP-CGG in Alum. 85d after the second immunization the mice were treated with tamoxifen, and a further 21d later immunized with NP-CGG in PBS or PBS alone and analyzed 5d after that, as shown in the time-line. Dot plots show surface binding of antigen (NIP) and expression of IgD on splenic non-T cells (CD4$^-$CD8$^-$), which are mainly B cells, and on plasma cells (CD138$^+$). Gates show switched antigen-specific cells (NIP$^+$IgD$^-$) and were used to determine data shown in Fig 4A.
